# *In situ* assessment of Mindin as a biomarker of podocyte lesions in diabetic nephropathy

**DOI:** 10.1371/journal.pone.0284789

**Published:** 2023-05-02

**Authors:** Ana Luisa Monteiro dos Santos Martins, Alexia Borges Bernardes, Verônica Aparecida Ferreira, David Campos Wanderley, Stanley de Almeida Araújo, José Rodrigues do Carmo Neto, Crislaine Aparecida da Silva, Régia Caroline Peixoto Lira, Liliane Silvano Araújo, Marlene Antônia dos Reis, Juliana Reis Machado

**Affiliations:** 1 Department of Pathology, Genetics and Evolution, Discipline of General Pathology, Institute of Biological and Natural Sciences of Federal University of Triângulo Mineiro, Uberaba, Minas Gerais, Brazil; 2 Institute of Nephropathology, Center for Electron Microscopy, Federal University of Minas Gerais, Belo Horizonte, Minas Gerais, Brazil; 3 Department of Bioscience and Technology, Institute of Tropical Pathology and Public Health, Federal University of Goiás, Goiania, Goiás, Brazil; Centre Hospitalier Sud Francilien, FRANCE

## Abstract

Diabetic nephropathy (DN) is the leading cause of chronic kidney disease and end-stage renal failure worldwide. Several mechanisms are involved in the pathogenesis of this disease, which culminate in morphological changes such as podocyte injury. Despite the complex diagnosis and pathogenesis, limited attempts have been made to establish new biomarkers for DN. The higher concentration of Mindin protein in the urine of patients with type 2 diabetes mellitus suggests that it plays a role in DN. Therefore, this study investigated whether *in situ* protein expression of Mindin can be considered a potential DN biomarker. Fifty renal biopsies from patients diagnosed with DN, 57 with nondiabetic glomerular diseases, including 17 with focal segmental glomerulosclerosis (FSGS), 14 with minimal lesion disease (MLD) and 27 with immunoglobulin A nephropathy (IgAN), and 23 adult kidney samples from autopsies (control group) were evaluated for Mindin expression by immunohistochemistry. Podocyte density was inferred by Wilms’ tumor 1 (WT1) immunostaining, while foot process effacement was assessed by transmission electron microscopy. Receiver operative characteristic (ROC) analysis was performed to determine the biomarker sensitivity/specificity. Low podocyte density and increased Mindin expression were observed in all cases of DN, regardless of their class. In the DN group, Mindin expression was significantly higher than that in the FSGS, MCD, IgAN and control groups. Higher Mindin expression was significantly positively correlated with foot process effacement only in class III DN cases. Furthermore, Mindin protein presented high specificity in the biopsies of patients with DN (p < 0.0001). Our data suggest that Mindin may play a role in DN pathogenesis and is a promising biomarker of podocyte lesions.

## Introduction

Diabetic nephropathy (DN) is one of the most important microvascular complications of diabetes mellitus (DM) [1,2] and is considered the leading cause of chronic kidney disease (CKD) and end-stage renal failure worldwide [3,4]. Approximately 30% to 40% of patients with type 1 and type 2 diabetes develop DN [5,6], which is histologically characterized by mesangial expansion, glomerular basement membrane (GBM) thickening and podocyte lesions caused by hyperglycemia, mechanical stress and increased levels of angiotensin II and transforming growth factor beta [7].

Podocytes are terminally differentiated cells with limited repair capacity. Thus, damage or podocyte loss leads to homeostasis dysregulation in the glomerular filtration barrier (GFB) and proteinuria [8]. Due to the importance of podocytes in GFB, studying biomarkers related to podocyte lesions and their involvement in the progression of DN is necessary. Mindin, also known as Spondin 2, is a member of the Mindin/F-spondin family of proteins, which is secreted from the extracellular matrix and was first identified on the basal lamina of zebrafish [9]. Murakoshi et al. identified increased SPON2 protein and RNAm expression in mice with diabetes and in the urine of patients with type 2 DM, suggesting that Mindin is a potential biomarker of podocyte lesions in DN [10].

Although the measurement of urinary components is affordable and noninvasive, the final diagnosis of suspected glomerulopathy is based on histologic findings in kidney biopsy. Thus, this study evaluated the *in situ* expression of Mindin protein in renal biopsies from patients with DN to understand its relevance in DN pathogenesis and its use as a biomarker of podocyte lesions.

## Materials and methods

### Study subjects

The study included 50 renal biopsies from patients previously diagnosed with DN at the Kidney Research Center, Federal University of Triângulo Mineiro (UFTM), Uberaba, Minas Gerais, Brazil. Of all patients, 28 were males (56%), and 22 were females (44%), with a mean age of 51.1 ± 13.5 years (median = 53 years; range 23–75 years). Six patients had type 1 DM (12%), 32 had type 2 DM (64%), and 12 cases (24%) were not described in the patient records. The history of DM was >10 years for 25 (50%) patients and 1–10 years for 12 subjects (24%), with an average course of 12.5±7.6 years.

The control groups consisted of 23 kidney autopsies from individuals whose cause of death was not related to infectious disease or previous renal disorders.

Kidney biopsies from patients diagnosed with nondiabetic glomerular diseases were included for comparison of Mindin *in situ* expression: 17 with focal segmental glomerulosclerosis (FSGS), 14 with minimal change disease (MCD) and 27 with immunoglobulin A nephropathy (IgAN). The patients were matched by sex and age with the DN and control groups.

This study was approved by the Ethics Committee (CEP) of UFTM (number: 3,001,006).

### Diabetic nephropathy diagnosis

DN was diagnosed after sample evaluation under light microscopy (LM), direct immunofluorescence (IF) and transmission electron microscopy (TEM). LM was used for DN classification according to the pathological classification of diabetic nephropathy: Class I—isolated glomerular basement membrane thickening and only mild, nonspecific changes by light microscopy; Class II—glomeruli classified as mild (IIa) or severe (IIb) mesangial expansion but without nodular sclerosis; Class III—at least one glomerulus with nodular increase in mesangial matrix (nodular sclerosis or Kimmelstiel–Wilson lesions); Class IV—more than 50% global glomerulosclerosis with other clinical or pathologic evidence that sclerosis is attributable to diabetic nephropathy [11]. Immune deposit analysis by IF was used to rule out the possibility of diseases associated with immunocomplex formation overlapping DN. Ultrastructural evaluation by TEM was conducted to rule out other diseases overlapping DN to evaluate foot process effacement and GBM thickness [12–14].

### Mindin (SPON-2) and WT1 immunohistochemistry

Immunohistochemistry was performed on 2-μm paraffin-embedded fragments using the Novolink nonbiotinylated polymer system (Novolink Polymer Detection System Kit, BL, UK, lot 6067432). The primary antibodies and assay conditions were applied as follows: (1) monoclonal mouse anti-human Wilms’ tumor 1 (WT1), Dako M3561, 1:500 and antigen recovery performed with citrate pH 6.0 buffer; (2) anti-SPON2 polyclonal antibody, Abcam Ab187920, 1:1000 and EDTA pH 9.0 buffer for antigen recovery.

Immunostaining quantification was performed from 40x magnification micrographs of all glomeruli observed in the biopsies of DN, FSGS, MCD and IgAN and 10 glomeruli from the control samples. Mindin expression was described as the percentage of labeled area relative to the total area evaluated, which was evaluated using the AxionCam ICc 5 (Zeiss®) interactive image analyzer system. WT1 expression was quantified using ImageJ 1.53 software, and the result was expressed as the cell density per glomerular area (podocyte/x10^6^ μm^3^), according to Venkatereddy et al. [15].

### Foot process effacement analysis

Foot process effacement was assessed by measuring the width in TEM images (7000x magnification) captured with a Zeiss EM-900 microscope. Images of all viable glomerular loops from two glomeruli were evaluated using ImageJ 1.53 software. The glomerular loop length (μm) was divided by the number of foot processes observed in the respective loop. The mean values of each DN sample mean underwent factor π/4 correction to normalize the presumed random variation of the section angle related to the long axis of the podocyte [16]. The mean foot process width (FPW) of each biopsy evaluated was expressed in nanometers (nm) [12].

### Statistical analysis

Statistical analyses were performed using GraphPad Prism version 7.0 software, with the level of significance set at 0.05. All data were submitted to the Kolmogorov‒Smirnov test. Then, the Mann‒Whitney (U) test or Kruskal‒Wallis (H) followed by Dunn’s posttest was used according to the number of groups. Correlation analysis was conducted using Spearman’s test (rS). The diagnostic performance (sensitivity/specificity) of the Mindin biomarker was tested by receiver operative characteristic (ROC) curves with their respective area under the curve (AUC) and 95% confidence interval (95% CI).

## Results

### Podocyte alterations in diabetic nephropathy

**Fig 1:**
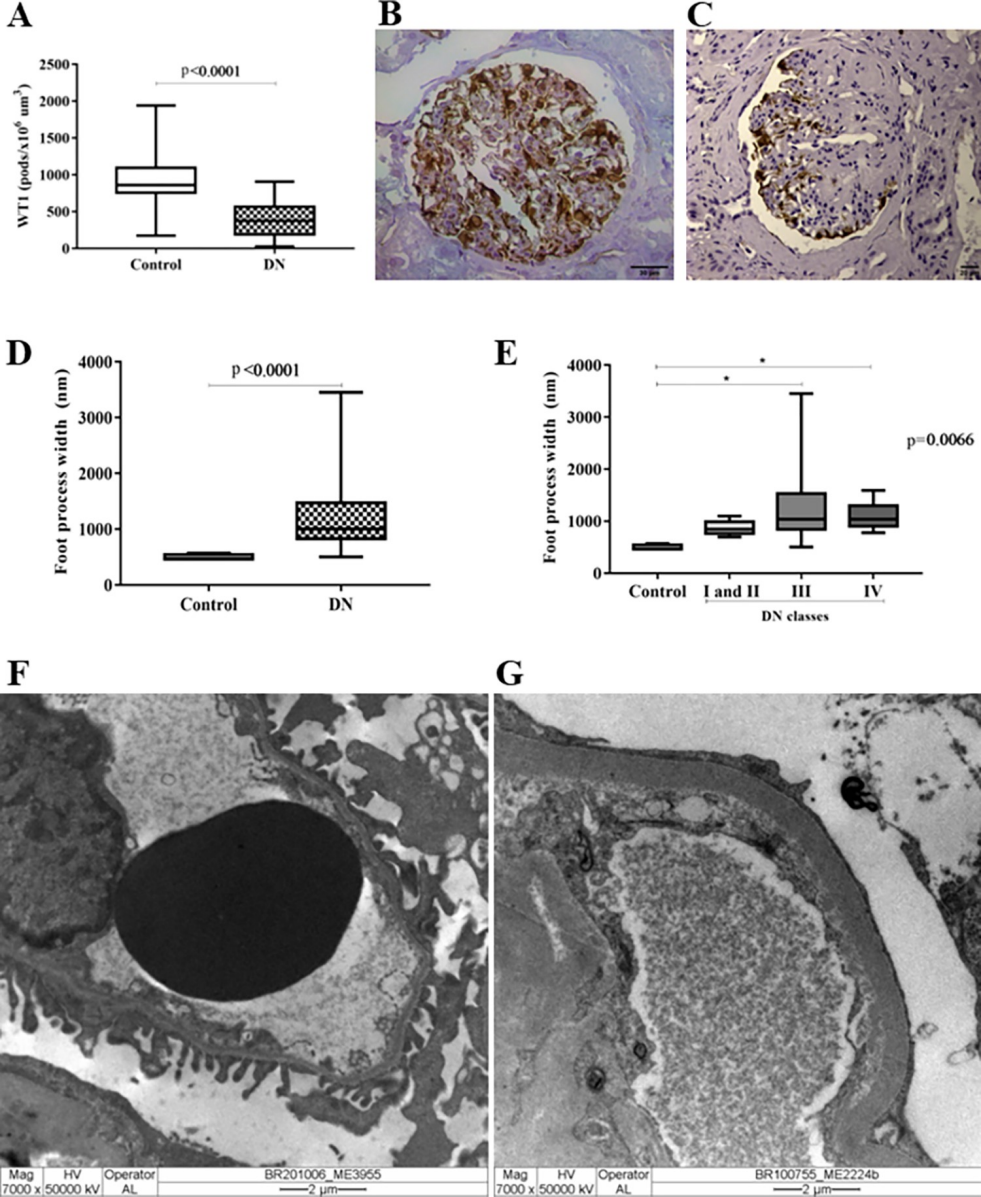
Podocyte density and foot process effacement in renal biopsies of diabetic nephopathy. (A) Significant reduction in podocyte density assessed by WT1 expression in DN cases compared to the control group. Mann‒Whitney test. (B) Representative biopsy of the control group showing high *in situ* WT1 expression in the glomerular compartment. (C) Representative biopsy of the DN group showing low WT1 expression in the glomerular compartment. (D) Foot process width in the control and DN groups. Mann‒Whitney test. (E) Foot process width in the control group and in different DN classes. Kruskal‒Wallis test followed by Dunn’s posttest. *p<0.05. (F) Normal pedicels under TEM in the control group. (G) Foot process effacement under TEM in the DN group.

The presence of podocytes in renal biopsies was analyzed by WT1 immunostaining. The DN group presented a significant reduction in podocyte density, which was demonstrated by the lower WT1 expression compared to the control group (p < 0.0001, U = 120, Fig 1A–1C). Since the number of podocytes was reduced in DN, we evaluated foot process effacement in DN cases, and we observed that FPW was higher in DN cases than in the control group, showing greater foot process effacement in the DN group (p < 0.0001, U = 1, Fig 1D). Although foot process effacement was present in all DN classes, it was significantly greater in classes III and IV than in the control group (p < 0.0066, H = 14.49, Fig 1E). Differences between the DN and control groups were evidenced by transmission electron microscopy analysis (Fig 1F–1G).

### Mindin protein as a biomarker of podocyte injury in DN

**Fig 2:**
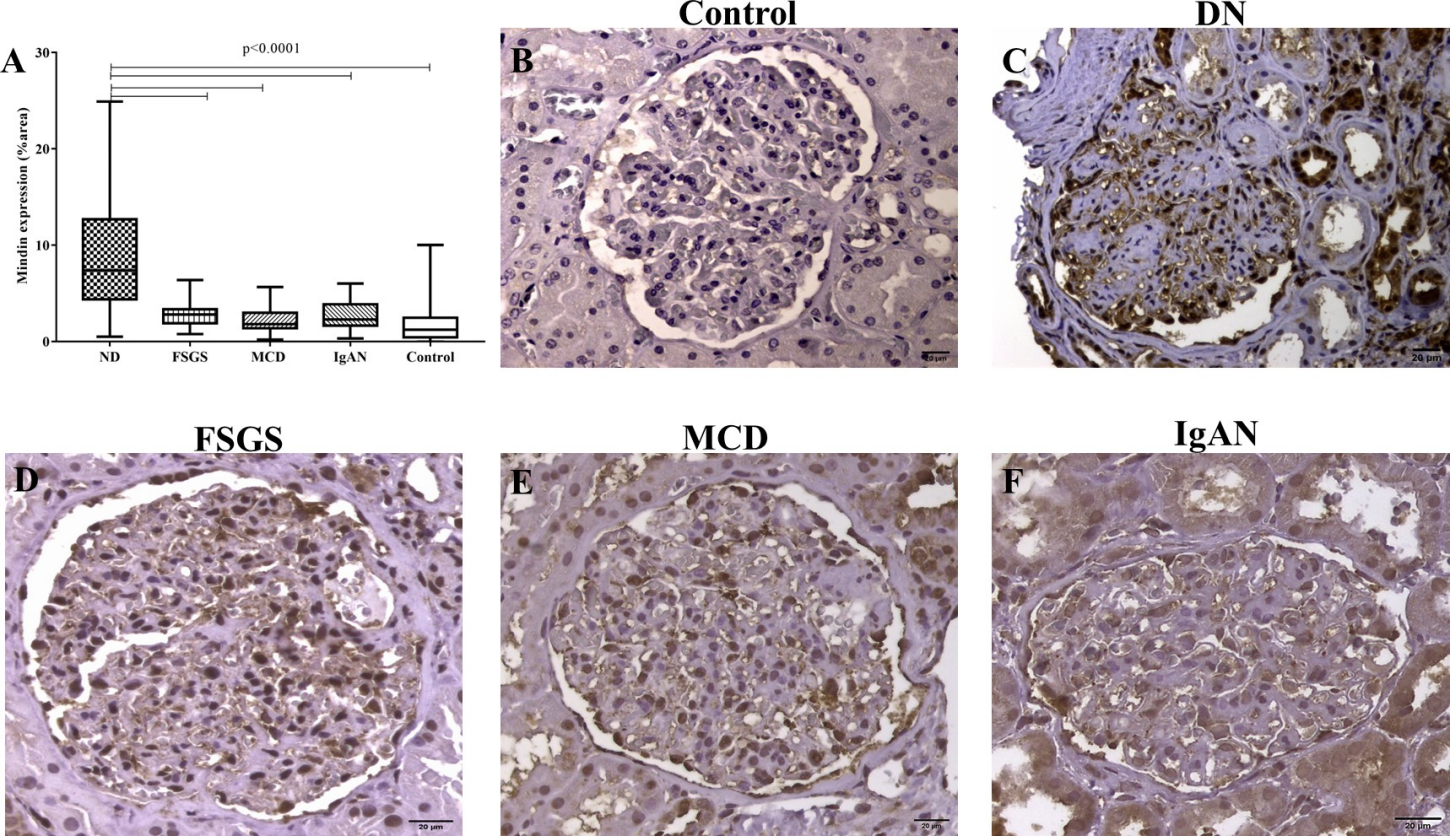
Mindin *in situ* expression in the glomerular compartment. (A) Comparison of Mindin *in situ* expression in DN cases, kidney biopsies from patients diagnosed with nondiabetic glomerular diseases and the control group. Kruskal‒Wallis test followed by Dunn’s posttest. p<0.05. (B) Low *in situ* Mindin expression in a representative biopsy of the control group. (C) Representative biopsy of the DN group showing high *in situ* Mindin expression. Representative biopsy of (D) FSGS, (E) MCD and (F) IgAN cases showing moderate *in situ* Mindin expression.

**Fig 3:**
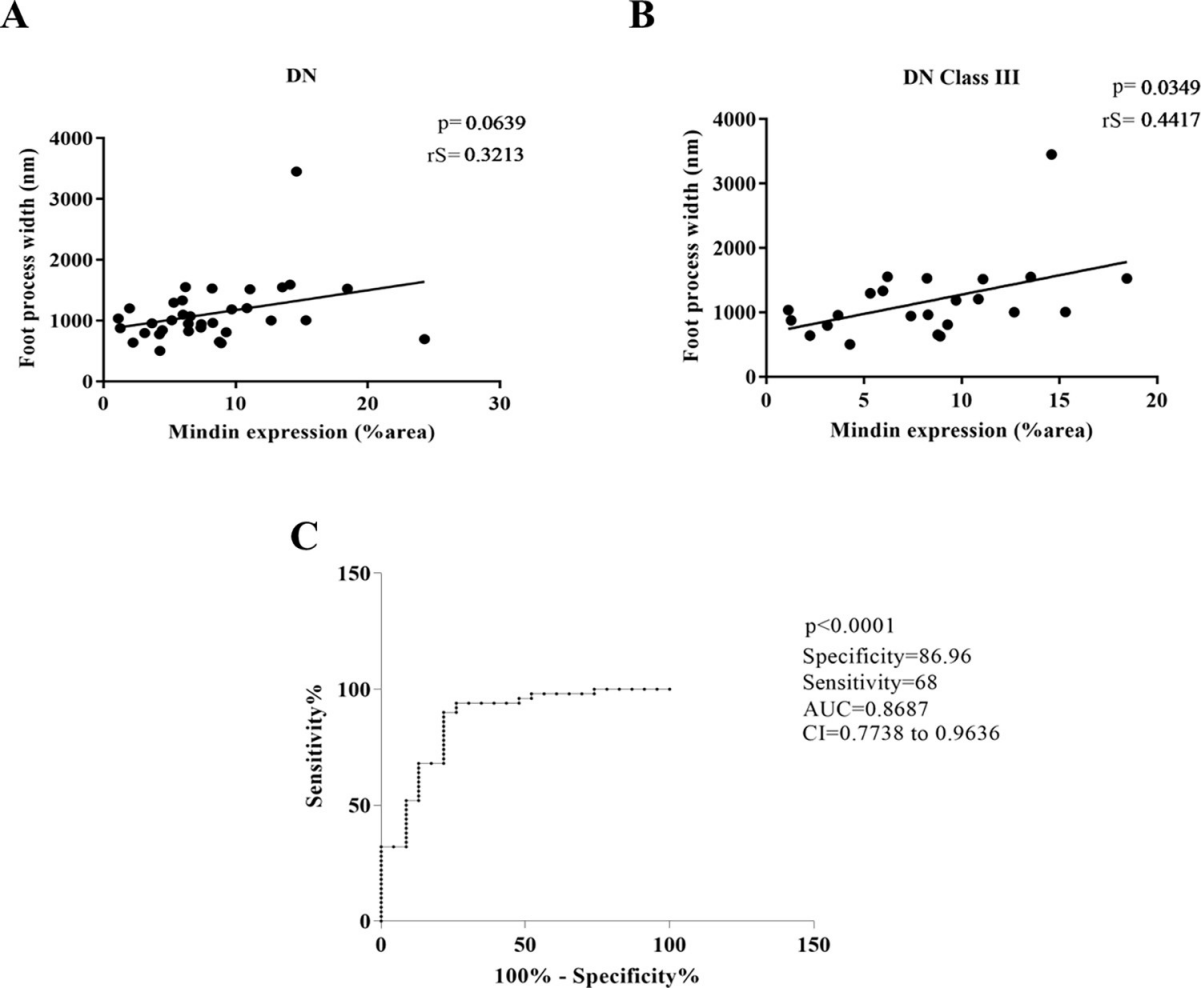
Mindin *in situ* expression and podocyte injury. (A) Trend of positive correlation between Mindin expression and foot process width in the DN group. Spearman’s correlation (rS). (B) Significant positive correlation between Mindin and foot process width in the class III DN group. Spearman’s correlation (rS). (C) The Mindin ROC curve in the DN group revealed high specificity and moderate sensitivity, showing Mindin protein as a biomarker of podocyte injury in DN.

As diabetic nephropathy morphologically presents a reduction in podocyte density and foot process effacement, Mindin *in situ* expression was assessed in renal biopsies to evaluate its potential association with podocyte injury. The glomeruli of patients with DN presented significantly higher expression of Mindin than the FSGS, MCD, IgAN and control groups (p < 0.0001, U = 55,81, Fig 2A–2F). Moreover, Mindin immunostaining showed a trend of positive correlation with foot process effacement (p < 0.0639, rS = 0.3213, Fig 3A), which was significant for the class III DN group alone (p < 0.0349, rS = 0.4417, Fig 3B).

**Fig 4:**
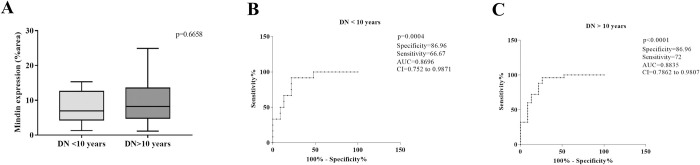
Mindin *in situ* expression and duration of DN. (A) Comparison of Mindin *in situ* expression in the DN < 10 years and DN > 10 years groups. Mann‒Whitney test. The Mindin ROC curve in the (B) DN group < 10 years and (C) DN > 10 years group revealed high specificity and moderate sensitivity.

Considering the higher expression of Mindin in DN biopsies and its correlation with FPW, we evaluated its usefulness as an *in situ* DN biomarker. The ROC curve revealed a sensitivity of 68% and specificity of 86.96% for Mindin, with an area under the curve (AUC) of 0.8687 (95% CI: 0.7738–0.9636, p < 0.0001, cutoff = 5.178% of stained area, Fig 3C). For classes I and II together, as well as class III alone, Mindin presented higher sensitivity (80% and 70.97%, respectively), with an AUC of 0.887 (95% CI: 0.7633–1.001, p = 0.0076) for classes I/II and an AUC of 0.8794 (95% CI: 0.7839–0.9749, p<0.0001) for DN biopsies of class III. Comparing Mindin expression with time of disease, we observed no significant difference between cases of DN <10 years and DN >10 years (p = 0.6658, U = 136, Fig 4A). However, the ROC curve revealed a sensitivity of 66.67% and specificity of 86.96% for Mindin, with an AUC of 0.8696 (95% CI: 0.752–0.9871, p = 0.0004, cutoff = 5. 597% of the stained area, Fig 4B) in DN cases <10 years and sensitivity of 72% and specificity of 86.96% for Mindin, with AUC of 0.8835 (95% CI: 0.7862–0.9807, p < 0.0001, cutoff = 5.178% of the stained area, Fig 4C) in DN cases >10 years.

## Discussion

Diabetic nephropathy is a serious complication of diabetes mellitus that progresses to end-stage renal failure. Despite its increasing prevalence worldwide, DN pathogenesis has not been completely described [17]. This study analyzed *in situ* Mindin expression in renal biopsies from patients with DN to explore associations with podocyte injury, as well as its potential application as an additional biomarker for DN morphological diagnosis. The majority of 50 biopsies evaluated were collected from male patients with a mean age of 51.1 years who were diagnosed with type 1 or type 2 DM more than 10 years ago. The subject profile was similar to previous reports involving patients with DN [18–20].

The DN morphological alterations were observed through different methods, such as immunohistochemistry and transmission electron microscopy. Considering that WT1 is a protein expressed in mature podocytes and is related to cell maintenance and differentiation [21], WT1 immunolabeling was conducted to assess podocyte density in renal biopsies. As expected, the DN cases presented significantly fewer labeled cells in glomeruli than the control group. WT1 is considered a podocyte biomarker and has been applied in other studies for the same purpose, both in biopsies and in experimental models [12,13,22]. In the DN context, the lower WT1 expression may be due to podocyte loss by cell detachment and apoptosis [12,23].

Foot process effacement is an ultrastructural podocyte finding in DN. Different protein alterations have been reported as the causes of this morphological podocyte change. Under diabetic conditions, experimental studies have demonstrated that foot process effacement is associated with increased compensatory actin stabilization and mutations in the gene that encodes the α-Actinin-4 protein [24]. The significant reduction in Nephrine expression was also described in renal biopsies from diabetic patients [25] and in podocytes from an animal model of early DN [26], both examples associated with foot process effacement. Moreover, the hyperglycemic environment of diabetes mellitus seems to suppress podocalyxin protein both *in vitro* [27] and *in situ* [28], contributing to podocalyxin uncoupling from the actin cytoskeleton and foot process effacement [29]. A third protein related to this change is podocalyxin, which is suppressed both *in vitro* [27] and *in situ*, as observed in streptozotocin-induced diabetic rats [28] and in biopsy samples from patients with diabetes [30]. In addition, foot process effacement is related to podocalyxin uncoupling from the actin cytoskeleton [29].

In addition to the lower WT1 expression in DN cases, this study observed significantly increased foot process effacement in DN renal biopsies compared to the control group. This finding corroborates Pagtalunan et al., who reported foot process effacement in DN [31].

Regarding Mindin immunostaining, the DN cases showed higher expression in the glomeruli than the control group. Previous studies have reported increased Mindin levels both *in situ* and in the urine of experimental DN models, as well as in the urine and blood of patients with DN [10,32]. However, to our knowledge, this is the first study to show *in situ* Mindin expression in renal biopsies from patients with DN. A well-documented mechanism explains the relationship between hyperglycemia, increased Mindin production and inflammatory cell recruitment in DN [14,33] and other pathological processes [34,35]. It has been shown that Mindin acts as an integrin ligand, promoting the recruitment of inflammatory cells [36]. Thus, in the stress environment caused by DM hyperglycemia, Mindin may bind to integrins, inducing cell recruitment and proinflammatory cytokine production, which can contribute to the maintenance of low-grade chronic inflammation present in DN.

We also demonstrated that the *in situ* expression of Mindin was significantly higher in DN than in other nondiabetic glomerular diseases that also course with podocyte alterations (GESF, DLM and IgAN) and the control group. Recently, it was observed that serum Spon2 levels were higher in patients with primary glomerular diseases than in control and nonglomerular kidney disease groups; however, there was no difference between the different glomerular diseases, demonstrating that serum Spon2 detection did not provide additional benefit in the differential diagnosis of nondiabetic glomerular diseases [37]. In view of these findings, our results suggest that Mindin is present in other glomerular diseases related to podocyte injury, but in DN, the *in situ* evaluation demonstrated that its expression is significantly higher, showing that Mindin may be related to podocyte injury caused by the hyperglycemic state. Since podocyte dysfunction is silent until proteinuria is detected [38], the increased expression of Mindin that will favor the early detection of injury in DN and its *in situ* evaluation may serve as a useful biomarker in the differential diagnosis and podocyte dysfunction.

Mindin staining showed a positive correlation with foot process effacement, including in the nodular glomerulosclerosis class of ND. Our findings corroborate with a study that demonstrated higher levels of Mindin in an animal model of diabetes and in the urine of patients with DMT2, suggesting Mindin as a biomarker of podocyte lesions in diabetes [10]. Furthermore the ROC curve analysis for Mindin revealed high specificity, which suggests that Mindin plays an important role in the pathogenesis of ND. Additionally, the ROC curve analysis for Mindin revealed high specificity independent of the time of disease, demonstrating that the expression of Mindin may be related to the early appearance of podocyte injury in ND. Thus, we believe that Mindin may be considered a promising biomarker of podocyte injury in DN.

Our study showed that in DN, the *in situ* expression of Mindin is significantly higher than in nondiabetic glomerular diseases and that it is closely related to podocyte injury. Thus, we additionally suggest that *in* situations in which the patient is submitted to renal biopsy collection by indication, and for some reason it is not possible to obtain a sample for analysis under MET, the research of Mindin *in situ* expression in patients with DN may contribute as an indicator of the presence of renal injury, specifically podocyte injury that is only possible to be evaluated by ultrastructural analysis, which may contribute to a complementary analysis of this biopsy.

## Conclusion

This study provides evidence that Mindin protein is expressed *in situ* in renal biopsies of patients with DN and was associated with foot process effacement, suggesting a role in disease progression and significant evidence of podocyte lesions. Considering the high specificity found in ROC curve analysis and the differential expression of Mindin in renal biopsies of patients with DN, we can also conclude that Mindin can be a possible podocyte lesion biomarker in DN.
